# Potential inequities in availability of care from breast care nurses: a qualitative study reporting the experiences and perspectives of women with metastatic breast cancer in Australia

**DOI:** 10.1186/s12913-022-08269-8

**Published:** 2022-07-22

**Authors:** Andrea L. Smith, Frances Boyle, Sophie Lewis

**Affiliations:** 1 The Daffodil Centre, University of Sydney a Joint Venture With Cancer Council NSW, Sydney, NSW Australia; 2 University of Sydney, Camperdown NSW 2050, Syndey, Australia; 3 Patricia Ritchie Centre for Cancer Care and Research, Mater Hospital, Sydney, Australia; 4 Faculty of Medicine and Health, University of Sydney, Sydney, Australia

**Keywords:** Metastatic breast cancer, Supportive care, Breast care nurse, Nursing, Survivorship, Patterns of care

## Abstract

**Background:**

International consensus guidelines recommend patients with metastatic breast cancer have access to a nurse experienced in the treatment of metastatic breast cancer. This study aimed to explore women’s experiences of supportive care from breast care nurses, including their perspectives on the role breast care nurses currently play in providing support to people with metastatic breast cancer.

**Methods:**

Multiple semi-structured qualitative interviews with 38 women with metastatic breast cancer in Australia. Data relating to nursing care were extracted and analysed using thematic analysis.

**Results:**

Three themes were identified: (1) feeling that supportive care needs are unrecognised; (2) confusion about role and relevance of breast care nurse to those with metastatic breast cancer; (3) care from metastatic breast care nurses (when available) was appreciated, valued and beneficial. Participants’ experiences differed in relation to ease of access to, amount of contact with, and level of care provided by breast care nurses. Contact and care ranged from little or none to comprehensive and ongoing. A key system-level challenge was that the diversity of diagnostic and treatment pathways for metastatic breast cancer meant that no systematic means existed to support routine or regular contact between breast care nurses and participants. Participants who did report having access to a specialised metastatic breast care nurse placed considerable value on the care received. For these participants, care from the breast care nurse extended and complemented care from the oncologist and included much needed psychosocial and practical support. For these participants, the breast care nurse assumed the role of key contact and care coordinator and was valued for their availability, accessibility and responsiveness. High levels of trust developed between patient and breast care nurse.

**Conclusions:**

Findings indicate that there may be gaps and inequities in supportive care in Australia for people diagnosed with metastatic breast cancer, a finding that supports earlier reports of limited access to breast care nurses for people with metastatic breast cancer in Australia. The specialised metastatic breast care nurse could potentially play a key role in addressing the high level of unmet supportive care needs and improve continuity of care for these patients.

## Introduction

Worldwide, more than 2 million people were diagnosed with breast cancer in 2018 [1]. Of the 0.6 million deaths from breast cancer globally each year, the vast majority are due to metastatic breast cancer [1, 2]. Metastatic breast cancer occurs when the primary breast cancer spreads beyond the breast and lymph nodes to distant sites such as the bones, liver, lungs or brain. In Australia, an estimated 9,000–12,000 people are living with metastatic breast cancer [3] of whom 3000 die every year [4]. Data from between 1995 and 2013 indicate that median survival in high-income countries such as Australia is 2–3 years, although in some subsets of patients extended survival up to 5 or even more years is now experienced [2]. Although considerable gains have been made in the treatment and prognosis of those with metastatic breast cancer, it remains incurable for almost all patients [2, 5].

Patients with metastatic breast cancer usually receive ongoing treatment often over many years to slow progression of their disease or to palliate symptoms. Patients typically progress through a number of ‘lines’ of treatment, moving from one line of treatment to the next as resistance develops to their current treatment regime until all suitable treatment options are exhausted [5]. Treatment options and outcomes vary widely, driven primarily by the metastatic breast cancer subtype and patient characteristics resulting in varied and complex pathways and trajectories for patients [6]. The lifelong nature of treatment, complexities of care and uncertain disease trajectory mean patients often report multifaceted psychosocial healthcare needs in addition to their clinical healthcare needs, resulting in unique challenges for clinical and supportive care services often over many years.

The goal of contemporary supportive care in cancer is to maximise physical and emotional care, support patients through cancer therapies, enhance their adherence to treatments, facilitate transitions of care, and prepare patients and their families for the challenges ahead [7]. Supportive care in cancer has traditionally focused on the initial diagnostic and treatment phases and on end-of-life care. Consequently, those living long-term with advanced or metastatic cancers often report unmet supportive care needs [8], in particular, high levels of informational, psychological, physical and functional unmet needs [8]. Similarly, for people living with metastatic breast cancer, international surveys have reported that beyond medical needs focused on addressing the cancer itself, the patients’ most important need was for support (emotional, family and social support) [9]. The importance of appropriate support for those with metastatic breast cancer has been recognised internationally and in Australia. International consensus guidelines state that every metastatic breast cancer patient should have access to a nurse experienced in the treatment of metastatic breast cancer [10, 11]. Cancer Australia’s 2019 Statement on Best Practice in Metastatic Breast Cancer states that every metastatic breast cancer patient should have access to a key contact person to support communication and coordination of patient-centred care [12]. Australia’s 2015 Optimal Care Pathway for People with Breast Cancer states that it is usually the breast care nurse who is responsible for care coordination, including ensuring continuity of care [13]. The updated 2021 second edition also states that patients should be offered a referral to a breast cancer nurse within seven days of definitive diagnosis and that the multidisciplinary team should include a metastatic breast cancer nurse or, if not available, a resident breast cancer nurse who cares for early and metastatic breast cancer patients [14].

As these guidelines and recommendations indicate, breast care nurses could potentially play an important role in supporting people with metastatic breast cancer [15–19]. Breast care nurses are specialist nurses with education and training in the management, treatment and follow-up of patients diagnosed with breast cancer [20–22]. The role of the breast care nurse specialist was developed in the UK and subsequently adapted and adopted internationally [15, 20–22]. The breast care nurse is a well-respected and well-established entity within breast care teams [23], and has been shown to impact positively on the overall quality of clinical care provided to patients with breast cancer [24]. However, to date the primary focus of these nurse specialists has been in providing care during the diagnostic and early-stage treatment phases [25, 26]. A 2010 UK survey of breast care nurses found little evidence that women with metastatic breast cancer were routinely allocated a breast care nurse, with more than half of nurses reporting that they felt that care for metastatic breast cancer patients was currently inadequate within their hospital [15]. These findings were corroborated in 2012 by the UK’s metastatic breast cancer pilot data collection project, which found that only 53% of patients were recorded as being referred to a clinical nurse specialist at the time of recurrent/metastatic diagnosis [27].

Currently limited data exist in Australia relating to patterns of care from breast care nurses for people with metastatic breast cancer. In 2002 an evaluation of care for women with breast cancer (*n* = 842) from breast care nurses (*n *= 4) in a hospital in Victoria, Australia reported that despite women with metastatic breast cancer making up 35% of the breast cancer caseload at the hospital they comprised only 7% of the population seen by the breast care nurses [28]. The gap in supportive care was more recently highlighted by Australia’s peak breast cancer advocacy organisation, Breast Cancer Network Australia (BCNA). BCNA’s 2015 national survey of women with metastatic breast cancer (*n* = 582) found that those with metastatic breast cancer had limited contact with breast care nurses, with one-third reporting no access at all to a breast care nurse. Of those who had had contact with a breast care nurse, more than half reported having seen the breast care nurse only once or very infrequently [29].

The aim of the current study was to explore women’s experiences of supportive care from breast care nurses, including their perspectives on the role breast care nurses currently play in providing support to people with metastatic breast cancer. For this study we have adopted the Multinational Association of Supportive Care in Cancer definition of supportive care in cancer, that is ‘the prevention and management of the symptoms and side effects of cancer and its treatment across the cancer continuum from diagnosis to end of life; it includes support for patients, their families, and their caregivers’ [30].

## Methods

In this qualitative interview-based study, an interpretive social constructionist approach was used to explore the experiences of women with metastatic breast cancer of care from breast care nurses [31]. Data were drawn from multiple semi-structured interviews with 38 women with metastatic breast cancer. The interviews were conducted as part of a larger research project investigating Australian women’s experiences of metastatic breast cancer and cancer care, as well as the experiences of health professionals who provide care and support. The research team included a qualitative cancer health services researcher (who has a lived experience of metastatic breast cancer), a breast cancer medical oncologist with expertise in the supportive care needs of people with metastatic breast cancer, and a qualitative health sociology researcher with expertise in health and metastatic breast cancer.

### Participants, sampling and recruitment

Eligible participants were women with a diagnosis of metastatic breast cancer. Although men can be diagnosed with breast cancer, they make up < 1% of breast cancer diagnoses and were therefore not the focus of this study. Purposive sampling and a range of community recruitment strategies were used to ensure participants from across Australia, at different stages in their metastatic breast cancer diagnosis, and from different healthcare settings were included. Participants were recruited via flyers, advertisements and presentations through cancer and breast cancer organisations and peak bodies, direct recruitment through clinicians, and snowball recruitment through study participants.

### Data collection

After written informed consent was obtained, an interview was conducted by one author either face-to-face in a location convenient to the participant (e.g., the participant’s home) or over the phone (e.g., if a participant lived in a regional area). The interviews were audio recorded, de-identified and transcribed in full. Each participant was allocated a pseudonym. Where possible, each participant was interviewed on a further two occasions over a 12-month period to capture how experiences and expectations of support from breast care nurses changed over time. The three interviews also allowed the interviewer to build rapport with participants. The follow-up interviews were conducted about 3 months apart guided by participants’ availability and how they were feeling. Data collection started in August 2017 and was completed in January 2020. Seven participants took part in one or two interviews due to ill health (*n *= 4) or being unable to be recontacted (*n* = 3).

The interviews were semi-structured, guided by an interview schedule that had been developed through review of the literature, discussions within the project team and consultation with a consumer representative with metastatic breast cancer. Topics included feelings and experiences of living with cancer as well as experiences of health services. Probes explored women’s experiences of care from different health professionals and in different settings. In addition, follow-up interviews explored how women’s experiences of living with metastatic breast cancer and their experiences of treatment, care and support had changed since the last interview. Probes included: 'Have there been any changes to the support and care you have received from health professionals?’ In follow-up interviews, we specifically asked all participants whether a breast care nurse had played a role in providing care or support. Detailed field notes were taken after each interview. In this current paper we report the data that relate to women’s experiences of supportive care from breast care nurses, including their perspectives on the role breast care nurses currently play in providing support to people with metastatic breast cancer.

### Data analysis

A constructionist approach to thematic analysis [32] was used to explore women’s experiences of treatment, care and support. First, interview transcripts were read and reread to organise qualitative data into descriptive categories related to women’s experiences of care and support by one author. Data related specifically to experiences of nursing care and support were then extracted, categorised and analysed thematically by a second author. Participants were categorised according to whether or not they reported receiving care from a nurse, a general breast care nurse or specialised metastatic breast care nurse since being diagnosed with metastatic breast cancer. Using inductive thematic analysis based on the constant comparative method [32, 33], open codes were first generated based on repeated readings of the interview data which were then organised into higher order themes. Themes identified in the data were developed and discussed with all authors to ensure consistency, and their relevance to the extant literature examined. Analysis was iterative, constantly moving from the specific to the more general, with the aim of producing more generalizable categories and explanations. This enabled us to identify commonalities and patterns across the large number of participants and interviews. NVivo was used to organise data and for initial coding. 

## Results 

Thirty-eight women, aged 36–74 years old, participated in the study (Table 1). Participants were recruited from all six Australian states. Most of the participants (*n* = 31; 82%) were between 40 and 59 years of age when diagnosed with metastatic breast cancer. Fourteen participants (37%) were diagnosed de novo metastatic; the remainder (*n *= 24; 63%) had had a previous diagnosis of early breast cancer. Ten (26%) had bone metastases only, 11 (29%) visceral metastases only, and 17 (45%) bone and visceral metastases, including 6 (16%) with brain metastases. Time since diagnosis of metastatic breast cancer varied from < 1 year to 23 years. Of the 38 participants, 23 reported contact with or care from a breast care nurse since their metastatic diagnosis. Of these 23 participants, 14 reported ongoing care from a breast care nurse (10 from a specialised metastatic breast care nurse; 4 from a general breast care nurse). The other 9 of the 23 participants reported one-off or fleeting contact with a breast care nurse rather than ongoing care. The remaining 15 of the 38 participants reported that they had not had any contact with nor received care from a breast care nurse since their metastatic breast cancer diagnosis. Most of the participants reported minimal changes to access and support from breast care nurses across the 12-month interview period.

**Table 1:** Participant characteristics (*n* = 38)

Characteristic	Number (%)
Age (years)	
< 40	1 (3)
40–49	3 (8)
50–59	20 (53)
60–69	12 (32)
≥ 70	2 (5)
Age when diagnosed with metastatic breast cancer (years)	
< 40	1 (3)
40–49	16 (42)
50–59	15 (39)
60–69	6 (16)
Time since metastatic breast cancer diagnosis (years)	
≤ 2	14 (37)
3–5	10 (26)
6–10	7 (18)
> 10	7 (18)
Type of metastatic breast cancer diagnosis	
Recurrence	24 (63)
De novo	14 (37)
Metastatic sites*	
Bone only	10 (26)
Visceral only	11 (29)
Bone and visceral	17 (45)

^*^ At first interview

Three themes were generated during the analysis: (1) feeling that supportive care needs are unrecognised; (2) confusion about role and relevance of the breast care nurse to those with metastatic breast cancer; and (3) care from metastatic breast care nurses (when available) was appreciated, valued and beneficial (Tables 2, 3, 4 and 5). The first two themes relate primarily to the experiences of the participants who *did not have access* to a breast care nurse or had only minimal contact with a breast care nurse. These two themes provide insights into potential gaps or inequities in supportive care for people with metastatic breast cancer. The third theme relates specifically to the experiences of participants *who had access* to breast care nurses. This theme provided insights into how people with metastatic breast cancer experience breast care nurse’s supportive care, and the value they place on this care.

**Table 2:** Themes and sub-themes

**Feeling that their supportive care needs are unrecognised**
Not being ‘visible’ in the healthcare system, therefore not being known to breast care nurse
Expectations that they ‘know the ropes’: assumptions that women with metastatic breast cancer know the system
Having unmet needs for information, support and care coordination
Needing support from someone other than oncologist
**Confusion about role and relevance of breast care nurse to those with metastatic breast cancer**
Differing expectations / confusion as to support breast care nurses could provide
Breast care nurses’ primary role is to provide care and support for those with early breast cancer
Not yet ‘ill enough’ to be offered / require supportive care from breast care nurse
**Care from metastatic breast care nurses (when available) was appreciated, valued and beneficial**
Valuing breast care nurses’ accessibility, availability and responsiveness
Filling a gap: coordinating care, providing much-needed information, emotional and practical support
Providing reassurance future needs will be met, especially towards end of life

**Table 3:** Feeling that supportive care needs are unrecognised

Sub-themes	Illustrative quotations
Not being ‘visible’ in the healthcare system, therefore not being known to breast care nurse	‘You kind of get missed, because metastatic doesn’t follow that traditional route, and quite a lot of us have quite different pathways and different medications.’ Heidi (diagnosed 5 years ago aged 48; interview 2) ‘I haven’t seen a breast care nurse since being diagnosed with secondaries’ Donna (diagnosed 6 years ago aged 46; interview 1) ‘[A breast care nurse] did come and see me at chemo and said something about sorry they didn’t know I was doing radiation, they would have come and seen me in radiation. But I didn’t know that.’ Kylie (diagnosed 3 years ago aged 46; interview 1)
Expectations that they ‘know the ropes’: assumptions that women with metastatic breast cancer know the system	‘I’ve only seen a breast care nurse once … she just came and asked me did I want any advice and I said no. I didn’t have anything to ask and I haven’t seen one since.’ Rita (diagnosed 5 years ago aged 47; interview 2) ‘I’d had my mastectomy and [the breast care nurse said], “Oh, you’re only secondary. You’re fine now,” I never saw her again. I said, “I’d like to go to a support group.” But nothing ever happened. Nobody cared … because I wasn’t primary diagnosed I got left behind.’ Christine (diagnosed 9 years ago aged 54; interview 1)
Having unmet needs for information, support and care coordination	‘When you’re in an appointment with an oncologist and there’s so much information sometimes, not always, it just would be good to know where you could go and spend five minutes talking to a nurse just to get clarification on stuff.’ Nicole (diagnosed 1 year ago aged 41; interview 1) ‘I think if the breast care nurses are mainly dealing with the surgical patients for early breast cancer, they need an alternative for women who are now facing a lifetime of dealing with the illness. Yeah, there should be some contact point, I think, at the hospital to deal with those people.’ Tammy (diagnosed 3 years ago aged 47; interview 1) ‘I think when you’re first diagnosed [with early breast cancer] you’re sort of allocated a breast care nurse. But I haven’t seen a breast care nurse since being diagnosed with secondaries. I’ve found that I’ve had to be quite proactive in getting what I want in terms of information about other services and things and I think if there was someone through the hospital system that could do that for you or with you, that that could be really useful.’ Donna (diagnosed 6 years ago; interview 1)
Needing support from someone other than oncologist	‘If you needed help, more than drugs and keeping you ticking [along], [the oncologists] just don’t have time. You need someone else to spend time with you regarding really sticky questions.’ Diane (diagnosed 7 years ago aged 50; interview 2) ‘I think with some oncologists I think they see things so much in a scientific way that they don’t really see the person or the woman.’ Nancy (diagnosed 5 years ago aged 58; interview 1)

### Theme 1: feeling that supportive care needs are unrecognised

Many participants reported a sense of being overlooked by the healthcare system, that they were not visible to the breast care nurses, and that their supportive care needs were unrecognised (Table 3). One of the issues that many participants talked about was how the metastatic breast cancer care pathway rarely brought them into contact with a breast care nurse and that there seemed to be no system for being allocated a nurse.

*One of the things was, because I didn’t have surgery first, I wasn’t allocated a breast care nurse and the public hospital I went to, and the nurses were lovely, but there wasn’t actually an allocated breast care nurse. So I would have gone two or three months without anyone explaining or showing me where I could go for information. Kara (diagnosed 8 years ago aged 48; interview 1)*

This was particularly problematic for participants such as Kara (above) who had been diagnosed with de novo metastatic breast cancer. These participants felt particularly disadvantaged as they had no previous experience of breast cancer and did not have an existing or prior relationship with a breast care nurse. For some it appeared that breast care nurses (and the healthcare system more broadly) assumed that a metastatic breast cancer diagnosis meant that you already knew about breast cancer and understood how the breast cancer healthcare system worked. Others such as Kylie who had had an earlier breast cancer diagnosis seemed frustrated by the perception that people diagnosed with metastatic breast cancer did not need support or that their supportive care needs were somehow less important than those of people with an early breast cancer diagnosis:

*Maybe the new people need [the breast care nurses] more ... maybe they think we’re old hands at it.* Kylie (diagnosed 3 years ago aged 46; interview 3).

Others commented that even when they did meet a breast care nurse, the breast care nurse appeared to be busy, to have little time in their working day to devote to them or else were unable to provide them with the type of support they needed. Offers of support arising from what were generally ad hoc encounters with breast care nurses were sometimes perceived by participants to be tokenistic. Some participants such as Nicole, below, drew comparisons between the care received from breast care nurses during their early breast cancer diagnosis and their subsequent metastatic diagnosis:

*The big difference that I’ve found between having early breast cancer and metastatic is back then you have really amazing nurse support. I think that’s because people are often having chemo or surgery. Whereas with advanced there seem to be fewer dedicated nurses about ... I feel like this time round if you really need help you really have to go and find it, rather than it being there.* Nicole (diagnosed 1 year ago aged 41; interview 1)

Many participants appeared to receive most of their care through their oncologist. Although participants talked highly of the care from oncologists, many also reported that gaps in care existed owing to the pressures of short appointment times, limited access to their oncologist, and a belief that the oncologist was time poor and unable to talk at length about treatment decisions or potential side-effects. This left participants sometimes feeling overwhelmed and with no-one to help explain what was going on and why.

*[Oncologists] they’re time poor ... So, even though I’m sure she would love to spend extra time with you, she can’t. She physically can’t do it. You go in there with your results and she’s focusing on totally your physical wellbeing. She hasn’t got time for your mental wellbeing.’* Diane (diagnosed 7 years ago aged 50; interview 2)

Diane, who had been living with metastatic breast cancer for seven years but who was only recently allocated a breast care nurse, went on to explain how the breast care nurse could complement and extend care from the oncologist. In the quote below, Diane explains how her breast care nurse provided support during difficult transitions such as disease progression:

*You need that debriefing to digest it and also to regroup. Sometimes you may want to just have a cry or just to talk to somebody ... If you’ve got questions about treatment and side-effects and that nitty-gritty stuff that you want to know that your oncologist can’t sit down and talk to you about because of time constraints.* Diane (diagnosed 7 years ago aged 50; interview 2)

Several participants, such as Nicole (previous page) reported that the absence of a breast care nurse had left them with no option but to proactively seek out information and support services themselves but they wondered how other people might cope, for instance, those with lower levels of health literacy or less able to navigate the healthcare system. There was concern that some patients may feel left behind or simply not receive the care they needed. This potential gap in services was reinforced by reports from other participants about how they were struggling with unmet supportive care needs, particularly the need for information, care coordination and navigation, emotional support, and help with managing relationships with family and friends.

### Theme 2: confusion about role and relevance of breast care nurse to those with metastatic breast cancer

Many participants expressed confusion about the role of the breast care nurse in providing supportive care to people with metastatic breast cancer (Table 4). For example, Janet reported unmet needs for information during her first interview (which was soon after being diagnosed with a metastatic recurrence of her breast cancer) but was not sure how a breast care nurse could potentially provide support:

*‘I feel that I don’t get answers to some of the questions that I want ans wered.’ … ‘No there’s no breast care nurse. In a way why would [there be]? It’s not about my breasts.’* Janet (diagnosed <1 year ago aged 60; interview 1)

**Table 4:** Confusion about role and relevance of breast care nurse to those with metastatic breast cancer

Differing expectations / confusion as to support breast care nurses could provide	‘‘The breast care nurse sat in [when I first saw the surgeon] and gave some information. But then further tests were done which showed that it had metastasised. So, there’s no breast care nurse. Anyway, look, I’m not sure now what a breast care nurse would do for me because I’ve taken a lot of it into my own hands. But I would hate to imagine what it’s like for a lot of women who are not that way inclined or don’t know the system.’ Rose (diagnosed 1 year ago aged 69; interview 2)
	‘I’ve never been offered [a nurse] or anything else that you could probably be offered. I don’t know what else you could be offered, but I’ve never been offered anything to complement or relate to what I’ve got, other than the actual oncologist and wha t he does.’ Vicki (diagnosed 11 years ago aged 48; interview 2)
	‘Look, I hear people talking about breast care nurses, and that may well be a place to go, but I’ve never known or been directed to a breast care nurse, or even really understand what their role is.’ Sharon (diagnosed 1 year ago aged 64; interview 2)
	‘I haven’t even seen [a nurse] … I haven’t had the need to, I suppose.’ Melanie (diagnosed 1 year ago aged 48; interview 3)
	‘There’s a link missing between when you’re diagnosed and what services are available to you. That’s what I think is missing. I think this is where the breast care nurses could have a more active role in it. You need an advocate to fight, to let you know what is available to you, because you don’t always know.’ Rebecca (diagnosed 4 years ago aged 47; interview 1)
Breast care nurses’ primary role is to provide care and support for those with early breast cancer	‘I think my final chemotherapy a breast care nurse at the hospital popped her head in, but she basically said, “I’m around, but I’m mainly dealing with people who have early breast cancer who are having surgery.”’ Tammy (diagnosed 3 years ago aged 47; interview 1)
	‘[Someone in our support group joked] “Well, [the breast care nurses] don’t need to pay attention to us because we’re going to die anyway.” I think there don’t seem to be enough, so they’re spending a lot of time with women with first stage.’ Rose (diagnosed 1 year ago aged 69; interview 2)
	‘My impression, and I could be totally wrong because I haven’t seen [a breast care nurse], but my impression of the breast care nurses is that they are very much sort of involved with that initial, your surgery, your initial chemo, making sure you’re okay, all that sort of thing. But I don’t know, I just think if you had a person who really understands metastatic breast cancer and all the services and things like that, that you could just go to with questions, yeah, would be really useful.’ Donna (diagnosed 6 years ago aged 46; interview 3)
	‘[I couldn’t] necessarily [ask] the nurses [questions] because they’re not trained—They’re oncology nurses, but they’re not specific. They used to have someone who was a breast care nurse, but she’d left. I could ask some general questions, but not specific questions. So no, there wasn’t anyone.’ Kara (diagnosed 8 years ago aged 49; interview 1)
Not yet ‘ill enough’ to be offered /require supportive care from breast care nurse	‘I’ve been offered no psychology, no counselling, nothing. It’s just been, go and fend for yourself. Go for chemo, go for radio, and go and sort yourself out. I’m wondering if that is on offer when it gets to a certain point. Because I’m walking around breathing and talking and looking like everybody else and I don’t have anything majorly wrong with me, as far as everyone’s concerned, if they look at me. But there’ll come a time when things do start going wrong and I think maybe the advanced breast care nurse will probably be advantageous then. But at the moment I’m not sure. I don’t know. I don’t know if there’s supposed to be a structure where you’re offered options and things. I just don’t know.’ Vicki (diagnosed 11 years ago aged 48; interview 2)

However, as Janet’s quotes from interviews 2 and 3 indicate, once Janet was allocated a breast care nurse this provided reassurance even though at this point in her diagnosis she did not feel the need to use the breast care nurse. Like many participants, Janet was aware that her need for support would increase with time.

*‘Yeah, I do feel as if I’ve got networks. I can ring up the oncologist office or a breast care nurse.’* Janet (diagnosed <1 year ago aged 60; interview 2)

*‘I do like knowing there’s a metastatic breast care nurse, even though I don’t [currently] use that service.’* Janet (diagnosed <1 year ago aged 60; interview 3)

Other participants were aware of a gap in care but were unsure who could best support them given they needed help with clinical, psychosocial and practical challenges, that the disease had both acute and chronic phases, and that their needs varied over time. Tammy wondered if what was needed was an alternative to the traditional breast care nurse as these women were *‘now facing a lifetime of dealing with the illness’* (diagnosed 3 years ago aged 47; interview 1); while Donna commented that: ‘*you’re sort of beyond the breast care nurse stage’* (diagnosed 6 years ago aged 46; interview 2). Donna and others suggested that what was currently missing was a care coordinator or nurse practitioner and that access to such support when needed could potentially improve the quality of the care they received and relieve some of the burden of living with metastatic breast cancer.

*One thing that I think is possibly lacking is someone, not non-medical but … someone to just be like a care coordinator. Someone who even maybe contacts you every three months to say, “How are you going? Is there anything you need? … I just think if you had some sort of nurse practitioner or someone else that was like a coordinator of your care that could refer you here, there, and everywhere if and when necessary or just if you’ve got questions and things.* Donna (diagnosed 6 years ago aged 46; interview 1)

Several participants commented that they had heard of specialised metastatic breast care nurses but did not know what they did or how they could access their services. Others said that they currently did not have access to a breast care nurse but wondered whether a breast care nurse would be available to them when their illness progressed. For example, when they were experiencing more serious symptoms, or when they transitioned to palliative care as is illustrated by the excerpt from Vicki below:

*I’m walking around breathing and talking and looking like everybody else and I don’t have anything majorly wrong with me, as far as everyone’s concerned, if they look at me. But there’ll come a time when things do start going wrong and I think maybe the advanced breast care nurse will probably be advantageous then.* Vicki (diagnosed 11 years ago aged 48; interview 2)

### Theme 3: care from metastatic breast care nurses (when available) was appreciated, valued and beneficial

Participants who did have access to a breast care nurse (usually a specialised metastatic breast cancer nurse) saw themselves as fortunate, commenting that they knew of other people with metastatic breast cancer who had not seen a breast care nurse (Table 5). They shared a strong sense of gratitude and appreciation for the supportive care they received from their breast care nurse, as is illustrated in the excerpts from Tess, Nancy and Rebecca, below.

*I’ve met a lot of women that can’t even get access to a breast care nurse. It’s horrific. We’re just very lucky at [our] hospital’.* Tess (diagnosed 1 year ago aged 61; interview 1)

*I’m fortunate because I have a fantastic nurse. She’s just such an amazing advocate for me ... She’s probably the only person that I can really talk to, because I feel that she’s the only one that really understands.* Nancy (diagnosed 5 years ago aged 58; interview 1)

*The breast care nurse can kind of debrief with you after [seeing the oncologist] and answer any questions that you have or if you’re left shell-shocked there is someone there that can sort of talk you down and help you out.* Rebecca (diagnosed 4 years ago aged 47; interview 1)

**Table 5:** Care from metastatic breast care nurse (when available) was appreciated, valued and beneficial

Valuing breast care nurses’ accessibility, availability and responsiveness	‘[The breast care nurse is] always saying, “Just let me know if there’s anything I can help with.” And I know obviously she’s really busy, but she totally manages to get back to me.’ Nicole (diagnosed 1 year ago aged 41; interview 3)
	‘You can’t get in touch with the oncologist all the time. They’re too busy saving lives. Her advanced nurse is fantastic. So, you can ring her. Obviously you can’t on the weekends, but you can ring her and get advice.’ Diane (diagnosed 7 years ago aged 50 years; interview 2)
Filling a gap: coordinating care, providing much-needed information, emotional and practical support	‘My oncologist is great, but you have to have appointments … When I was feeling down, I messaged [the breast care nurse] about that and she gave me a couple of names for a psychologist. So, because of her, I was able to get onto the person I’m seeing now, and if I have issues I can just email her. When I’m having Herceptin every three weeks she often will just come down and touch base, which is good to have that continuity … So yeah, that’s terrific, to actually have someone. One person, not an organisation, but one person that knows what I’ve gone through or what I’m going through and who can refer me and has the knowledge to actually point me in the right direction and help me, is terrific.' Kara (diagnosed 8 years ago aged 49; interview 2)
	‘[The breast care nurse] breaks it down into plain English. If you’ve gone for an appointment and [the oncologist] is talking in their medical jargon and that sort of thing and you’re not really understanding and they’re trying to rush you through the appointment because they’ve got 10 other patients behind you.’ Rebecca (diagnosed 4 years ago aged 47; interview 1)
Providing reassurance future needs will be met, especially towards end of life	‘I do like knowing … there’s a metastatic breast care nurse, even though I don’t [currently] use that service … Just to see that and know that [the metastatic breast care nurse] is there.’ Janet (diagnosed < 1 year ago aged 60; interview 3)
	‘Having a metastatic breast care nurse has been amazing, because I know that I now have a resource. Because you can’t do that with your oncologist. They’re busy. You can do it when you meet them. But now knowing that I have a nurse who I can say, “I’ve got this.” Kara (diagnosed 8 years ago aged 49; interview 1)

As Rebecca’s quote indicates, participants placed considerable value not just on the metastatic breast cancer nurses’ in-depth and specialised knowledge of metastatic breast cancer but also on their availability and accessibility. Having access to someone who was approachable who had sufficient time to spend with them during key points in their disease trajectory, such as receiving bad news or changes in treatment, was crucial. Providing clarification and reassurance appeared to be an important part of the metastatic breast cancer nurse role, with many participants reporting that their metastatic breast cancer nurse helped them to deal with the emotional and existential concerns of living with metastases.

Participants also valued the metastatic breast cancer nurses’ understanding of how the health and social care systems worked as this was something that many reported difficulty navigating. Participants said that their breast care nurse often became involved in care coordination by putting them in contact with other health and supportive care services, and in advoca ting for them within and beyond the hospital system. Several participants, including those who had access to a breast care nurse but had not yet felt the need to contact them, reported that they valued the sense of reassurance and comfort afforded by simply knowing there was someone there to support them now, and in the future, who would be there to walk with them until the end.

## Discussion

This study reveals a desire among participants for continuous care and support from a specialised contact person such as a breast care nurse. However, access to and experiences of supportive care from breast care nurses varied widely and did not always align with patients’ expectations nor with recommendations, indicating potential gaps and inequities in supportive care for people with metastatic breast cancer in Australia. In particular, care received by participants did not always align with national recommendations, including those in Australia’s recently updat ed Optimal Care Pathways for People with Breast Cancer [14].

The Australian Optimal Care Pathways outline key principles of care, quality standards and support needs for 18 cancers, including breast cancer. Optimal Care Pathways are not legislated and are not mandatory in Australia but have been endorsed as government policy [34]. The Optimal Care Pathway for People with Breast Cancer recommends that patients should be referred to a breast cancer nurse within seven days of definitive diagnosis. They also state that it is usually the breast cancer nurse who is responsible for coordination of the patient’s care and for ensuring continuity of care [14]. The gaps in care highlighted in the current study, in particular access to a breast care nurse, reinforce the importance not only of these Optimal Care Pathways but of the need to develop implementation strategies that will ensure these principles of care are applied systematically across all stages in the breast cancer care continuum, including metastatic breast cancer.

A key finding is that many of the participants reported that they did not have a relationship with a breast care nurse. Only about a third of participants reported being supported by a breast care nurse, which was typically a specialised metastatic breast care nurse. Of the remaining participants, a small number reported receiving support from a non-breast care nurse such as a clinical trial nurse, a chemotherapy nurse or a radiotherapy nurse. When available, care from a breast care nurse was highly regarded and complemented and extended care from the oncologist. These gaps and inequities in care are potentially concerning given the increasing attention on cancer survivorship in those with advanced cancer [35, 36], the rapidly expanding number of people living long-term with advanced cancer [37], and the focus on making cancer care patient-centred, comprehensive and integrated [38–40].

Our study highlighted that the varied, typically non-surgical, care pathways that participants followed when diagnosed with metastatic breast cancer meant that many did not routinely come into contact with breast care nurses. Similar findings were reported in a 2010 survey of breast care nurses in the UK [15]. Findings from this survey indicated that the assessment and care pathways from metastatic diagnosis through to treatment were unstructured and ill-defined, making it problematic for breast care nurses to identify where and when patients with metastatic disease would present. The authors concluded that if the breast care nurses were unable to identify the patient group it was not possible to identify, assess and address the needs of these patients [15]. It would appear that as in the UK, in Australia breast care nurses provide care primarily during the diagnostic and early-stage breast cancer treatment phases, despite those with metastatic breast cancer having higher supportive care needs [15, 41].

In our study, several participants speculated that the lack of support from breast care nurses might be because breast care nurses may not have sufficient time in their working day to look after those with metastatic breast cancer. This patient perspective confirms what breast care nurses in the UK and Australia have reported about their role, that is, the majority of their time is spent supporting patients with early breast cancer [15, 42] leaving them little time in their working day to care for those with metastatic breast cancer [43]. Furthermore, many breast care nurses report feeling ill-equipped to address the multidimensional needs of those with metastatic breast cancer [15, 42]. The 2010 UK study reported that although many of the breast care nurses would like to offer a better service to people with metastatic breast cancer, many felt limited by staffing numbers within their teams, a lack of knowledge about metastatic disease, its treatment and manifestations, the challenge of caring for patients with progressive cancer and inadequate time within their working day [15]. The UK study concluded that improvements to service delivery in breast cancer care would require the provision of dedicated resources and appropriate training and support for those required to undertake these roles.

Our findings also reveal several contradictions. Some participants rationalised that the lack of supportive care from breast care nurses might be because they were not yet ‘ill enough’ to require support. In contrast, others thought that the lack of care might be due to nihilistic attitudes towards metastatic disease. We believe these contradictions are reflective of larger issues in cancer care. First, the need to recognise that advances in treatment for metastatic cancer have resulted in a growing population of patients with ‘treatable but not curable’ cancer who are living for years instead of months [44, 45]. These patients are highly unlikely ever to be cured, often have high supportive care needs, but are not yet in need of palliative care. The supportive care needs of these patients are not easily addressed by a cancer care delivery system geared towards supporting patients through initial diagnosis and treatment and end-of-life or palliative care [46]. Second, there is a widespread lack of integration of oncology and supportive care, which results in a focus on treating the disease (the cancer) rather than the patient with the disease [47].

This study found that participants who did have access to one of Australia’s few specialised metastatic breast cancer nurses placed considerable value on this service, in particular the breast care nurse’s accessibility, responsiveness, knowledge and ability to coordinate care and advocate on their behalf. The current study reinforces and extends the findings of a 2009–2010 evaluation of the implementation of the specialised metastatic breast cancer nurse role in a cancer centre in New South Wales, Australia [25] which reported strong support from patients and healthcare professionals for the continuation and expansion of the specialised metastatic breast cancer nurse role. However, it was only recently in 2019 that funding became available for a limited number (*n* = 21) of specialised metastatic breast care nurses in Australia [48]. 

### Strengths and limitations

While exploratory in nature, a strength of this qualitative study is that it reports first-hand the voices and experiences of people with metastatic breast cancer in Australia. High levels of validity and rigour were achieved through: a pilot-tested interview schedule to ensure consistency of questions; multiple interviews over 12 months to build rapport; participation by most participants in all three interviews; input from the research team into theme development; and attention to the ways in which the researchers participated in the construction of particular narratives (reflexivity) within the context of an interview [49].

A potential limitation of our study is closely linked to the key finding – that despite there being a desire for continuous care and personal support from a specialised contact person, relatively few (*n* = 14) participants reported having a relationship with or knowledge of the role of the breast care nurse. While qualitative studies rely on rich and thick data collection and analysis, we would argue that this lack of data is also important. What is not being talked about by participants provided us with insights into potential gaps in care for this subpopulation of cancer survivors [44]. This limitation also meant limited data were available that related to how the breast care nurse helped to meet the supportive care needs of people with metastatic breast cancer. This is an important area that could be explored in future qualitative research. A further limitation is that qualitative studies collect in-depth data rather recruiting a representative sample and generating generalisable findings. It is therefore likely that other people with metastatic breast cancer in other areas of Australia might report different experiences of supportive care from breast care nurses. It should also be acknowledged that the provision in 2019 by the Australian Federal Government of funding for 21 specialised metastatic breast cancer nurses may have reduced the gap in service provision highlighted in the current study [48]. Furthermore, the development in 2020 of the first Australian model of nursing care for metastatic breast cancer may have had a positive impact on experiences of care from breast care nurses [50].

## Conclusions

In the past 20 years much has been written about the unmet needs of those with metastatic breast cancer [9, 15, 16, 18, 42, 51, 52]. The continued gaps in supportive care from breast care nurses identified in our study indicate that further attention should be given to implementing what has been recommended internationally as best practice in breast cancer care [10, 11], in particular, the recommendation that all patients with metastatic breast cancer have access to a nurse experienced in the treatment of metastatic breast cancer [10, 11]. More broadly, there also needs to be a greater appreciation at the health system and healthcare provider level of the distinctiveness of metastatic breast cancer from early breast cancer, in particular the unique supportive care needs of those with metastatic breast cancer and how these vary at different points in the disease trajectory, and during acute and chronic phases of illness [53]. Future research is needed to understand the value and role of the specialised metastatic breast cancer nurse from the perspective of the metastatic breast cancer nurse, the woman with metastatic breast cancer, and her family and to understand the availability of breast care nursing care for those with metastatic breast cancer in Australia.

## Data Availability

The data that support the findings of this study comprise de-identified transcripts. Data are available from the authors upon reasonable request and with permission of the University of New South Wales Human Research Ethics Committee.

## Abbreviations

BCNA: Breast Cancer Network Australia
UK: United Kingdom
